# Auxin homeostasis in maize (*Zea mays*) is regulated via 1-*O*-indole-3-acetyl-*myo*-inositol synthesis at early stages of seedling development and under abiotic stress

**DOI:** 10.1007/s00425-022-04058-z

**Published:** 2022-12-20

**Authors:** Anna Ciarkowska, Patrycja Wojtaczka, Jacek Kęsy, Maciej Ostrowski

**Affiliations:** 1 Department of Biochemistry, Nicolaus Copernicus University, Lwowska 1, 87-100 Torun, Poland; 2 Department of Plant Physiology and Biotechnology, Nicolaus Copernicus University, Lwowska 1, 87-100 Torun, Poland

**Keywords:** Abiotic stress, Auxin, Cold stress, Drought stress, IAA, IAA ester conjugates, IAGlc synthase, IAInos synthase, Phytohormones, *Zea mays*

## Abstract

**Main conclusion:**

Indole-3-acetyl-*myo*-inositol biosynthesis is regulated during maize seedling development and in response to drought and cold stress. The main purpose of this pathway is maintenance of auxin homeostasis.

**Abstract:**

Indole-3-acetic acid (IAA) conjugation to *myo*-inositol is a part of a mechanism controlling free auxin level in maize. In this work, we investigated changes in the indole-3-acetyl-*myo*-inositol (IAInos) biosynthesis pathway in 3-d- and 6-d-old maize seedlings and germinating seeds as well as in seedlings subjected to drought and cold stress to evaluate a role of this pathway in maize development and stress response. In germinating seeds, activity of the enzymes involved in IAInos biosynthesis remains unchanged between 3-d- and 6-d-old material but increases in coleoptiles and radicles of the seedlings. Under cold stress, in germinating seeds and in coleoptiles, activity of the enzymes decreases and increases, respectively; however, it does not entail changes in auxin level. In drought-exposed germinating maize seeds, totally diminished activities of IAInos synthesis pathway enzymes resulted in almost twofold increase of free IAA content. Similar increase of auxin level was observed in radicles of drought-subjected seedlings together with lack of catalytic activity of the first enzyme of the pathway. Exogenous IAInos has no effect on the level of non-enzymatic antioxidant, ascorbate. It has also either no effect on the protein carbonylation and lipid peroxidation, or it affects it in a similar way as exogenously applied IAA and *myo*-inositol, which are products of IAInos hydrolysis. Thus, IAInos biosynthesis pathway acts in maize development and stress responses by regulation of free IAA concentration, as IAInos itself does not appear to have a distinct role in these processes.

## Introduction

Indole-3-acetic acid (IAA), a main naturally occurring auxin, regulates cell division, elongation and differentiation; hence, almost every process in a plant throughout its entire life cycle is affected by this phytohormone (Woodward and Bartel 2005). Plant processes regulated by IAA include tropic responses, such as gravitropism, primary root and hypocotyl elongation, lateral root initiation and apical dominance (Bajguz and Piotrowska 2009). Plant response to IAA is mainly dependent on the free auxin concentration, thus, maintenance of IAA homeostasis is of vital importance for proper plant growth and development (Normanly 1997). Disruption of auxin homeostasis results in altered plant phenotype. Reduced free IAA content leads to dwarf plants with increased branching and long primary roots with few lateral roots (Jackson et al. 2002; Woodward and Bartel 2005; Yu et al. 2019). On the other hand, IAA overproducing mutants typically exhibit elongated hypocotyls, short primary roots and abundance of lateral roots (Woodward and Bartel 2005; Kim et al. 2013; Ke et al. 2015).

Biological activity typical for auxins is exhibited only by free IAA. Local concentration of the phytohormone is regulated by several mechanisms, such as de novo biosynthesis, degradation, transport from the area of high auxin accumulation level to the destination site and synthesis or hydrolysis of IAA conjugates (Ludwig-Müller 2011; Korasick et al. 2013).

IAA conjugates are a predominant form of auxin in all examined plant species (Sztein et al. 1995, 2000). Free IAA can constitute only up to 25% of the entire auxin pool, though its level is usually much lower and is species- and tissue-specific (Ludwig-Müller 2011). The IAA carbonyl group can form an amide bond with an amino group of amino acids, peptides or proteins. Such IAA amide conjugates are synthesized by enzymes from Gretchen Hagen3 (GH3) family and they predominate in dicotyledonous plants such as *Arabidopsis thaliana* or *Pisum sativum* (Bajguz and Piotrowska 2009; Korasick et al. 2013). Maize (*Zea mays*) and other monocotyledonous plants mostly conjugate IAA to sugars and alcohols by forming an ester bond between the auxin carbonyl group and the hydroxyl group of the second molecule, though IAA amide conjugation pathway also functions in these plants.

Synthesis and hydrolysis of IAA conjugates are important for modulation of free auxin level and thus are regulated by phytohormones, including IAA itself, and in response to various stress conditions (Ludwig-Müller 2011). Even though their function is not completely understood, it is believed that IAA conjugates play other significant roles besides regulating its concentration by being temporary auxin storage forms. Some amide conjugates, IAA-aspartate (IAA-Asp) and IAA-glutamate, are intermediates of auxin degradation (Korasick et al. 2013). Ester conjugates are considered to participate in IAA transport (Bajguz and Piotrowska 2009). IAA conjugates do not display typical auxinic activity and hence they used to be considered biologically inactive. However, a number of studies suggest that conjugated forms of IAA have a specific role in fruit ripening (Bӧttcher et al. 2010), responses to pathogens (Domingo et al. 2009; González-Lamothe et al. 2012), and abiotic stresses such as salinity, cadmium, drought or high temperature (Oetiker and Aeschbacher 1997; Tognetti et al. 2010; Ciarkowska et al. 2016; Ostrowski et al. 2016; Pavlović et al. 2018).

As mentioned above, monocotyledonous plants, such as maize, mostly accumulate IAA in form of its ester conjugates. In the endosperm of maize kernels, half of the conjugated auxin consists of IAA-*myo*-inositol and its glycosides (Ueda and Bandurski 1974; Jensen and Bandurski 1994).

The IAA ester conjugates synthesis pathway in maize begins with the formation of 1-*O*-indole-3-acetyl-β-D-glucose (1-*O*-IAGlc, IAGlc) (Michalczuk and Bandurski 1980):

$$ {\text{IAA}}\, + \,{\text{UDP}} - {\text{glucose}}\, \leftrightarrow \,{1} - O - {\text{IAGlc}}\, + \,{\text{UDP}}{.} $$

This reaction is catalyzed by UDPG-dependent IAA glucosyltransferase (1-*O*-IAGlc synthase). 1-*O*-IAGlc is an energy-rich molecule and acts as a donor in a subsequent transacylation reaction which is catalyzed by an enzyme of a serine carboxypeptidase-like (SCPL) acyltransferases family, 1-*O*-(indole-3-acetyl)-β-D-glucose: *myo*-inositol indoleacetyl transferase (IAInos synthase) (Kęsy and Bandurski 1990; Kowalczyk et al. 2003). IAInos synthase catalyzes the transfer of IAA moiety from 1-*O*-IAGlc to *myo*-inositol with the formation of IAA-*myo*-inositol (IAInos):

$$ {1} - O - {\text{IAGlc}}\, + \,myo - {\text{inositol}}\, \to \,{\text{IAInos}}\, + \,{\text{glucose}}{.} $$

IAInos synthesized in this reaction can either stay in this form or undergo further modifications into its glycosides (Corcuera and Bandurski 1982; Corcuera et al. 1982). It can also be hydrolyzed to free IAA and *myo-*inositol. Interestingly, the latter reaction is catalyzed by IAInos synthase, a bifunctional enzyme responsible for both synthesis and hydrolysis of the conjugate (Kowalczyk et al. 2003). IAInos is considered to function as a storage form of IAA, thus biosynthesis and hydrolysis of this conjugate directly affect the level of active auxin in plant tissues.

Auxin, as a major phytohormone, plays a crucial role in regulation of plant growth at every stage of plant development. In our previous study (Ostrowski et al. 2020), we have determined that IAA-*myo*-inositol biosynthesis pathway plays a role in the regulation of auxin level during the development of maize seeds. Here, we report how the expression and activity of enzymes of this pathway change at the early stages of maize seedling development. As the exposition of seedlings to abiotic stresses can greatly affect crop yields, we have also examined IAA-*myo*-inositol biosynthesis pathway under drought conditions and cold stress.

## Materials and methods

### Plant material and treatments

Maize (*Zea mays*) cultivar Golden Midget (Bio Planet, Leszno, Poland), commonly cultivated in European countries, was used as a plant material. Maize seeds were soaked in distilled water for 24 h. Plants were grown on Petri dishes lined with filter paper in darkness at 25 °C for 3 or 6 days. Coleoptiles, radicles and germinating seeds (whole seed) were excised from 3-d- or 6-d-old seedlings and subjected to analysis. To initiate drought conditions, 5-d-old plants were transferred to 20% (w/v) polyethylene glycol (PEG) 6,000 and grown in darkness at 25 °C for 24 h. For cold stress, 5-d-old plants were transferred to 10 °C and grown in darkness for 24 h.

Levels of ascorbate, protein carbonyl groups and lipid peroxidation were determined in 6-d-old maize coleoptiles and radicles. Before the analysis, 5-d-old seedlings were transferred to distilled water (control conditions), 10 μM IAA, 10 μM IAInos or 10 μM *myo*-inositol and grown for additional 24 h. IAInos was synthesized by Professor Stanisław Kowalczyk from Department of Biochemistry, Nicolaus Copernicus University, Poland, according to the method of Nowacki et al. (1978) and kindly provided by him.

### Tissue homogenates preparation for enzymatic activity assays

Maize germinating seeds, coleoptiles and radicles were homogenized (1 g tissue: 5 mL buffer for seeds and coleoptiles, 1 g tissue: 10 mL buffer for radicles) with 25 mM Tris–HCl buffer, pH 7.5, using mortar and pestle. The homogenates were centrifuged at 10,000 g for 10 min at 4ºC (Sigma Sartorius 3 K 30 Centrifuge, 12,154 rotor, Sartorius, Göttingen, Germany). The supernatant fluid was used for enzymatic activity assays.

### IAGlc synthase (EC 2.4.1.121) activity assay

Enzymatic activity of IAGlc synthase was determined in a total volume of 8 μL containing 74.1 mM HEPES buffer, pH 7.4, 10.9 mM UDPG, 5.8 mM IAA, 592 Bq [2’-^14^C]IAA (2.035 GBq mmol^−1^; Hartmann Analytic GmBH, Braunschweig, Germany), 18 mM D-gluconic acid lactone and 3.6 mM MgCl_2_, with 3 μL of the supernatant fluid from tissue homogenates. The reaction was stopped after 30 min incubation at 30 °C by drying 4 μL of aliquots on Silica Gel F_260_ TLC plate (Merck, Darmstadt, Germany). TLC was performed using ethyl acetate: n-butanone: ethanol: water (5: 3: 1: 1, by vol.) as a solvent. For indole compounds visualization, the plate was stained with van Urk-Salkowski reagent (Ehmann 1977). Bands corresponding to IAGlc were excised and placed in a vial with 2 mL EcoLite ( +) scintillation fluid (MP Biomedicals, Irvine, CA, USA). Radioactivity level was measured in Wallac 1409 liquid scintillation counter (Wallac Oy, Turku, Finland).

### IAInos synthase (EC 2.3.1.72) activity assay

Enzymatic activity of IAInos synthase towards IAInos biosynthesis was determined in a total volume of 8 μL containing 25.2 mM HEPES buffer, pH 7.4, 12 mM UDPG, 6.4 mM IAA, 592 Bq [2’-^14^C]IAA (2.035 GBq mmol^−1^; Hartmann Analytic GmBH), 15 mM *myo*-inositol, 18 mM D-gluconic acid lactone, 4 mM MgCl_2_ and 3 μU of recombinant IAGlc synthase, with 3 μL of the supernatant fluid from tissue homogenates. The reaction was stopped after 30 min incubation at 30 °C by drying 4 μL of aliquots on Silica Gel F_260_ TLC plate (Merck). TLC was performed using ethyl acetate: n-butanone: ethanol: water (5: 3: 1: 1, by vol.) as a solvent. For indole compounds visualization, the plate was stained with van Urk-Salkowski reagent (Ehmann 1977). Bands corresponding to IAInos were excised and placed in a vial with 2 mL EcoLite ( +) scintillation fluid (MP Biomedicals). Radioactivity level was measured in Wallac 1409 liquid scintillation counter (Wallac Oy).

### RNA isolation and quantitative reverse transcription (qRT-PCR) analysis

Total RNA was extracted from the maize germinating seeds, coleoptiles and radicles using RNA Extracol (EURx, Gdańsk, Poland). RNA samples were pretreated with RNA-se free DNase I (Thermo Scientific, Waltham, MA, USA) to remove any contaminating genomic DNA. First-strand cDNA synthesis was performed using 1 μg of RNA with RevertAid First Strand cDNA Synthesis Kit (Thermo Scientific) according to the manufacturer’s instructions. PCRs of 20 μL were prepared using the LightCycler 480 SYBR Green Master I (Roche Diagnostics, Basel, Switzerland) with 50 ng of template cDNA. IAA ester conjugates synthesis pathway genes expression was analyzed using following gene-specific primers: *ZmIAGlc* (IAGlc synthase encoding gene; GenBank NM_001111856.1)—F: 5ʹ-CAGGGACACATGAACCCCAT-3ʹ; R: 5ʹ-TCGGCAGTCCTCTGGATGAA-3ʹ; *ZmIAIn* (IAInos synthase encoding gene; GenBank BT016677.1)—F: 5ʹ-AGTGGTTCGCTGAATACGCA-3ʹ; R: 5ʹAGTTGATCTTGACCCCACCG3ʹ. To normalize sample variance, *ZmFPGS* gene (folylpolyglutamate synthase gene, GenBank NM_001158593.1) was used as the endogenous control (primers—F: 5ʹ-CCTCAGAGATTGCTCCCACG-3ʹ; R: 5ʹ-TGTTCACGCTCTGAAGGTGG-3ʹ). Real-time thermo-cycling was performed using LightCycler 480 (Roche Diagnostics) with the following cycling conditions: 95 °C for 5 min, followed by 45 cycles of 95 °C for 10 s, primer annealing (55 °C for *ZmIAGlc*, 60 °C for *ZmIAIn* and *ZmFPGS*) for 20 s, and 72 °C for 20 s. The specificity of each reaction was confirmed on the basis of the melting curve. The relative expression of analyzed genes was calculated with LightCycler 480 Software by the ΔCt method and normalized using *ZmFPGS* as a reference gene. Reference gene was chosen based on research by Manoli et al. (2012) who evaluated a number of maize genes used as a reference for qPCR analysis.

### Determination of free IAA level

Level of free IAA was determined by modified QuEChERS (quick, easy, cheap, effective, rugged and safe) method (Perestrelo et al. 2019) using internal deuterated standard and followed by liquid chromatography tandem mass spectrometry (LC–MS/MS). Approximately 100 mg of tissue was ground in liquid nitrogen using mortar and pestle and further homogenized with 1.6 mL of 80% (v/v) acetonitrile containing 1 mM 2,6-di-tert-butyl-4-methylphenol and 5% (w/v) formic acid. d-2-IAA (OlChemIm, Olomouc, Czech Republic) was used as an internal standard based on Lu et al. (2015), Porfίrio et al. (2016) and Zhang et al. (2020).

After addition of 10 ng of d-2-IAA, the homogenates were agitated (170 rpm) overnight at 4 °C. 60 mg of MgSO_4_ and 20 mg of NaCl were added to the homogenates. Samples were subsequently vortexed and agitated (170 rpm) for 30 min at room temperature. The homogenates were centrifuged at 20,000*g* for 10 min at 4 °C (Sigma Sartorius 3 K 30 Centrifuge, 12,154 rotor) to separate water and acetonitrile phases. The upper acetonitrile phase was transferred to new tubes and then evaporated to dryness under nitrogen stream at 45 °C. The remaining residue was suspended in 1 mL of 1 M formic acid and loaded onto pre-conditioned (washed with 2 mL of 100% (v/v) methanol and 2 mL of 1 M formic acid) Bakerbond SPE column (Avantor Performance Materials, Center Valley, PA, USA). After sample application, the columns were washed with 2 mL of 1 M formic acid and the retained analytes were eluted with 0.5 mL of 80% (v/v) methanol. Elutes were subsequently lyophilized and suspended in 100 μL of 40% (v/v) methanol with 0.1% formic acid (w/v) and analyzed with LC–MS/MS.

Analysis was performed on a Shimadzu Nexera XR UHPLC system (Shimadzu, Kyoto, Japan) with Ascentis Express C-18 column (2.7 μm, 100 × 2.1 mm, Supelco, Bellefonte, PA, USA) as a stationary phase that was kept at 35 °C.The mobile phase consisted of (A) 0.1% formic acid and (B) methanol with 0.1% formic acid delivered as a binary gradient at a flow rate of 0.4 mL min^−1^. The gradient started at 40% B, then was raised linearly to 90% B during the next 4 min, and then to 100% B during the next 2 min. Next, the mobile phase was held at 100% for 1 min before equilibrating the column for 3 min with the initial concentration of 40% B. The injection volume was 2 μL. The LC was interfaced with a triple quadrupole mass spectrometer (LCMS-8045, Shimadzu) and operated in positive mode. The LC–MS/MS was operated in the positive mode, with the electrospray as the ionization source. Multiple reaction monitoring (MRM) acquisition was done by monitoring the 176.20/130.30 and 178.20/132.30 m/z transitions for IAA and d2-IAA, respectively. The peak area of the diagnostic product ion 130.30 and 132.30 under optimized conditions was used for quantification. Data acquisition was performed with LabSolutions software 5.8 (Shimadzu).

### Determination of ascorbate level

Plant material was homogenized with 5% (w/v) trichloroacetic acid (TCA), using a mortar and pestle (1 g tissue: 2 mL TCA for coleoptiles, 1 g tissue: 10 mL TCA for radicles). The homogenates were centrifuged at 10,000*g* for 10 min at 4 °C (Sigma Sartorius 3 K 30 Centrifuge, 12,154 rotor). The supernatant fluid was used for the ascorbate level assay.

The reaction mixture contained 0.27 mL of homogenate, 1.37 mL of phenanthroline solution (0.075% (w/v) phenanthroline, 4.8% (w/v) sodium acetate and 0.85% (v/v) acetic acid) and 0.28 mL of freshly prepared 1% (w/v) FeCl_3_. The blank sample contained distilled water instead of homogenate. The samples were vortexed and incubated at room temperature for 1 h. The ascorbate concentration was measured spectrophotometrically (UVmini-1240 UV–vis Spectrophotometer, Shimadzu) at 512 nm and calculated using a calibration factor. For the calibration curve, the reaction mixture contained 10, 30, 50, 70 or 100 μM ascorbate instead of the homogenate. Ascorbate level was expressed as nmol per gram of fresh weight (nmol g^−1^ FW).

### Determination of lipid peroxidation level

Level of malondialdehyde (MDA), a product of membrane fatty acids peroxidation, was determined as a marker of lipid peroxidation.

Homogenates were prepared as for ascorbate level analysis. The reaction mixture contained 0.5 mL of homogenate, 0.5 mL of 15% (w/v) TCA and 0.5 mL of 0.37% (w/v) thiobarbituric acid (or distilled water for blanks). The samples were vortexed, incubated at 95 °C for 10 min and cooled in ice. The MDA level was measured spectrophotometrically (UVmini-1240 UV–Vis Spectrophotometer, Shimadzu) at 535 nm and calculated using molar absorption coefficient *ε* = 156 mM^−1^ cm^−1^ and expressed as nmol per gram of fresh weight (nmol g^−1^ FW).

### Determination of protein carbonyl group level

Plant material was homogenized (1 g tissue: 2 mL buffer for coleoptiles, 1 g tissue: 10 mL buffer for radicles) with 50 mM phosphate buffer, pH 7.5 containing 2 mM β-mercaptoethanol, using a mortar and pestle. The homogenates were centrifuged at 10,000*g* for 10 min at 4 °C (Sigma Sartorius 3 K 30 Centrifuge, 12,154 rotor). The supernatant fluid was used for the assay. Level of protein carbonyl groups was assayed as described by Ostrowski et al. (2016) and expressed as nmol mg^−1^ protein.

### Protein concentration

Protein concentration was determined by Bradford method (Bradford 1976) using albumin as a standard.

### Statistical analysis

All experiments were performed in three biological replicates, each in three technical repetitions. All data are presented as mean ± SD for three biological repetitions of each experiment (*n* = 3). All statistical analyses were performed using the Student’s *t* test.

## Results

### Changes in IAInos biosynthesis pathway during maize seedling development

Auxin acts as a regulator of plant growth and developmental processes thus its local concentration in plant needs to be strictly controlled. One of the mechanisms responsible for managing IAA level is conjugation of the phytohormone resulting in loss of its auxinic activity (Casanova-Sáez et al. 2021). In monocots, such as maize, the main IAA conjugation pathway is a two-step biosynthesis of IAInos (Bajguz and Piotrowska 2009). First, IAA is conjugated to glucose and subsequently the auxinic moiety is transferred to *myo*-inositol. Both reactions are catalyzed by two enzymes of transferases family, IAGlc synthase and IAInos synthase, respectively (Michalczuk and Bandurski 1982). Taking into account the importance of IAInos biosynthesis pathway in maintaining auxin homeostasis, we have investigated both steps of said pathway at early stages of maize development. To compare potential changes in IAInos biosynthesis during early maize development we have chosen 3-d-old and 6-d-old seedlings as a plant material. We have analyzed transcription level and activity of both synthases involved in IAInos synthesis, as well as free IAA level, in coleoptiles and radicles of maize seedlings as well as in the seeds (whole seed) from which the seedlings emerged.

**Fig. 1:**
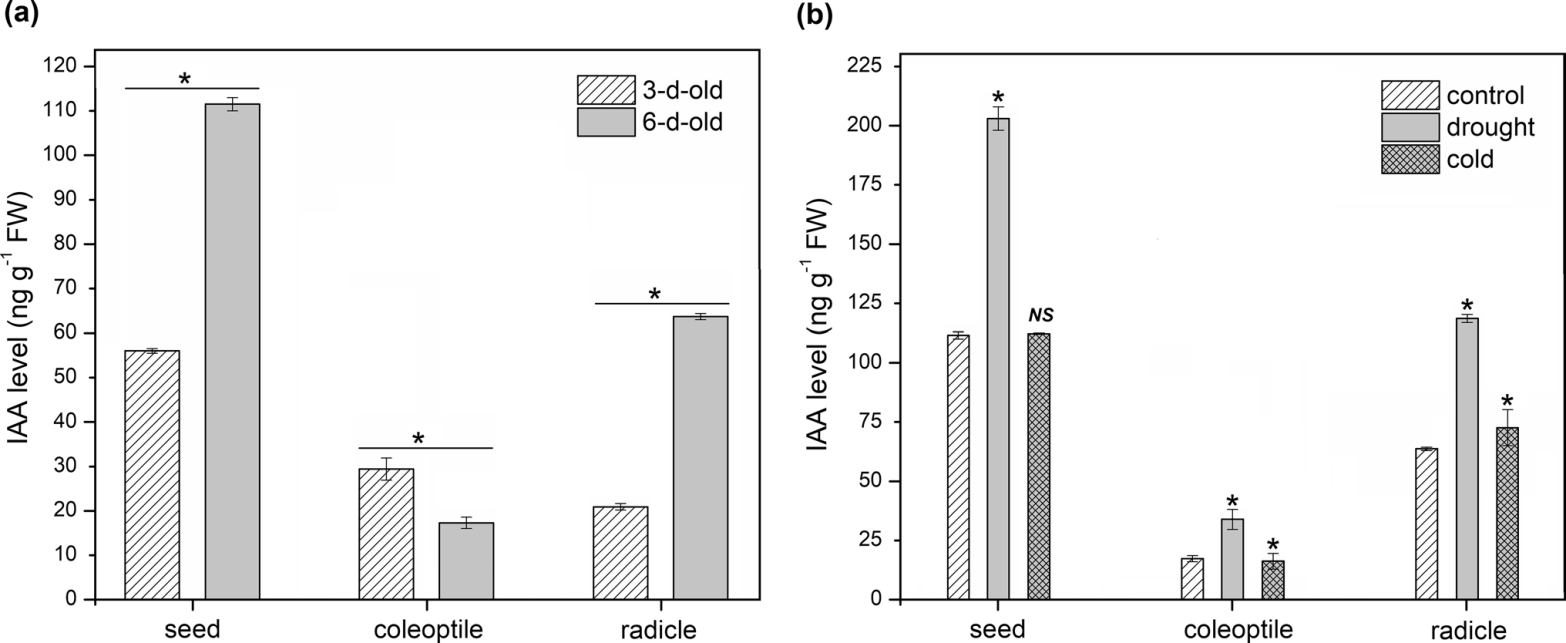
Free IAA concentration in maize determined by LC–MS/MS. **a** In 3-d- and 6-d-old maize germinating seeds, coleoptiles and radicles. **b** In 6-d-old maize germinating seeds, coleoptiles and radicles of control seedlings and maize subjected for 24 h to cold and drought stress. Data are expressed as mean ± SD for 3 biological repetitions of each assay (*n* = 3). Statistical significance is indicated by *, *P* < 0.05; *NS*, not significant (*P* > 0.05) (*t* test)

Results of IAA analysis by LC–MS/MS approach are shown in Fig. 1a. The highest free IAA level was characteristic for seeds, being 56 and 111.5 ng g^−1^ FW in 3-d- and 6-d-old material, respectively. Over time, free IAA profile changes in both coleoptiles and radicles. For maize coleoptiles, level of free IAA decreases 1.7-fold in 6-d-old seedlings compared to 3-d-old coleoptiles. However, in older radicles level of free IAA is threefold higher than in 3-d-old material.

**Fig. 2:**
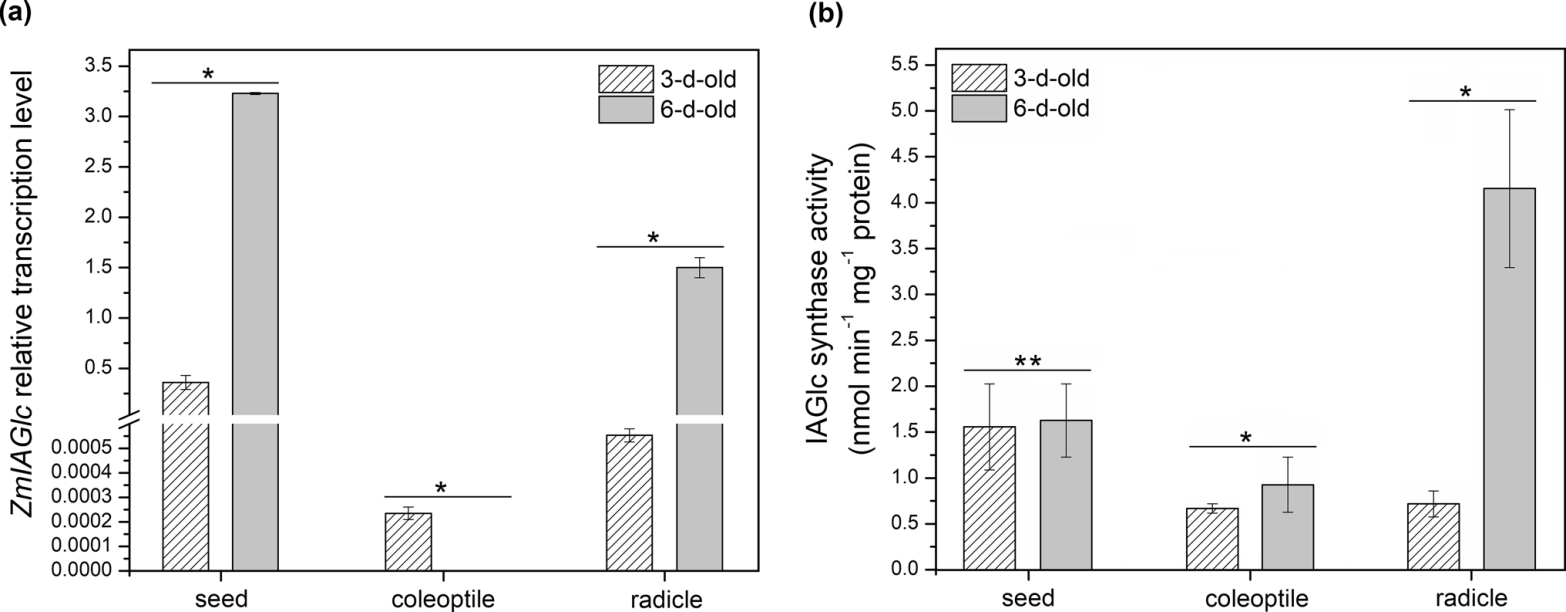
IAGlc synthase in 3-d- and 6-d-old maize germinating seeds, coleoptiles and radicles. **a** Expression level of *ZmIAGlc* determined by quantitative RT-PCR analysis. **b** Enzymatic activity of IAGlc synthase. Data are expressed as mean ± SD for 3 biological repetitions of each assay (*n* = 3). Statistical significance is indicated by *, *P* < 0.05; **, *P* < 0.01 (*t*-test). No bar visible in the graph means there was no transcript or activity detected in the material

IAGlc synthase catalyzes conjugation of IAA to glucose. As a result, 1-*O*-IAGlc is formed and can be utilized as a substrate for further IAA conjugation process (Michalczuk and Bandurski 1982). Using the qPCR method, we have analyzed relative expression of IAGlc synthase encoding gene (*ZmIAGlc*) in 3-d-old- and 6-d-old maize coleoptiles and radicles as well as in seeds from which the young seedlings emerged. *ZmIAGlc* transcript level is shown in Fig. 2a. The highest relative expression level was detected in 6-d-old radicles (1.5) and seeds (3.23). High level of *ZmIAGlc* transcript was also present in 3-d-old seeds (0.36). On the other hand, in 3-d-old coleoptiles and radicles, expression level was low, being 2.53 × 10^–4^ and 5.35 × 10^–4^, respectively. 6-d-old coleoptiles were the only material where no *ZmIAGlc* transcript was detected.

We have also analyzed the enzymatic activity of IAGlc synthase. The results are presented in Fig. 2b. IAGlc synthase was the most active in 6-d-old radicles (4.16 nmol min^−1^ mg^−1^ protein) which corresponds to high level of the transcript. High catalytic activity of the enzyme was also detected in both 3-d (1.56 nmol min^−1^ mg^−1^ protein) and 6-d-old (1.63 nmol min^−1^ mg^−1^ protein) germinating seeds which is also in consistency with high relative expression of *ZmIAGlc* gene in this material. Thus, over time IAGlc activity was maintained on similar level in seeds despite ninefold difference in the transcript level. In 6-d-old maize coleoptiles, IAGlc synthase exhibited enzymatic activity of 0.93 nmol min^−1^ mg^−1^ protein even though no *ZmIAGlc* transcript was detected. It suggests that the enzyme is still present in the coleoptiles with no active expression of the gene.

**Fig. 3:**
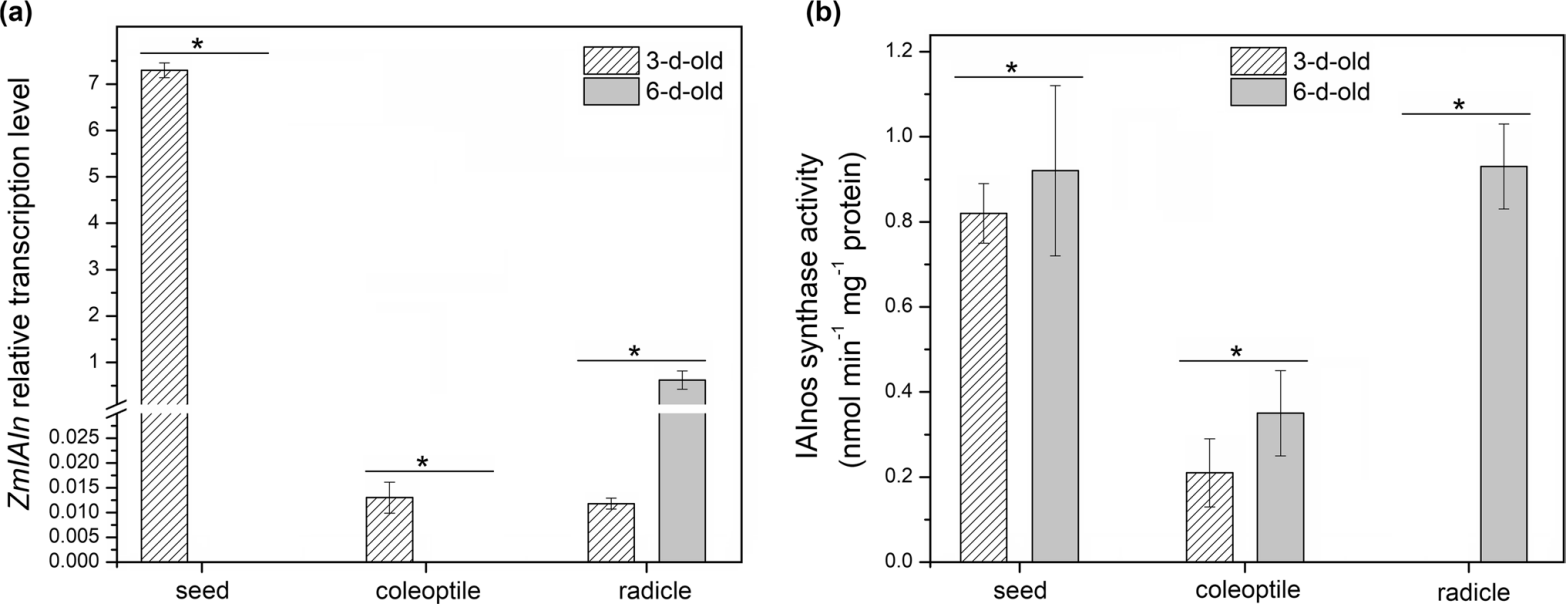
IAInos synthase in 3-d- and 6-d-old maize germinating seeds, coleoptiles and radicles. **a** Expression level of *ZmIAIn* determined by quantitative RT-PCR analysis. **b** Enzymatic (IAInos biosynthesis) activity of IAInos synthase. Data are expressed as mean ± SD for 3 biological repetitions of each assay (*n* = 3). Statistical significance is indicated by *, *P* < 0.05 (*t* test). No bar visible in the graph means there was no transcript or activity detected in the material

IAInos synthase catalyzes final step of IAInos biosynthesis which is a transfer of IAA from 1-*O*-IAGlc to *myo*-inositol. We have performed the same expression and activity analyses as for IAGlc synthase. No transcript of IAInos synthase encoding gene (*ZmIAIn*) was detected in 6-d-old coleoptiles and seeds (Fig. 3a); however, the enzyme is active in said material (Fig. 3b). IAInos synthase activity is one of the highest in 6-d-old seeds (0.92 nmol min^−1^ mg^−1^ protein). As shown in Fig. 3b, IAInos synthase displays high catalytic activity also in 3-d-old seeds (0.82 nmol min^−1^ mg^−1^ protein) and 6-d-old radicles (0.93 nmol min^−1^ mg^−1^ protein). It corresponds to high *ZmIAIn* transcript level which is 7.3 and 0.62, respectively (Fig. 3a). Low relative transcription was characteristic for 3-d-old coleoptiles and radicles being 1.3 × 10^–2^ and 1.18 × 10^–2^, respectively. Low transcription level is reflected in no catalytic activity of IAInos synthase in 3-d-old radicles.

### Changes in IAInos biosynthesis pathway in maize seedlings under drought and cold stress

As IAA is one of the main regulators of plant growth and development, its level is also crucial for plant adaptation to environmental stress under which the plant needs to focus more on survival instead of growth (Korver et al. 2018). Thus, we have also investigated IAInos synthesis pathway in young maize seedlings exposed to two kinds of abiotic stress: drought and cold. The pathway was investigated in coleoptiles and radicles of 6-d-old maize seedlings as well as in the seeds (whole seed) from which the seedlings emerged.

Free IAA level was the highest in seeds and the lowest in coleoptiles of the maize seedlings (Fig. 1b). In all examined material, the level of the phytohormone was not affected by cold stress but increased under drought. Compared to the control, in drought-subjected seedlings, free IAA level was 1.8-fold, 2-fold and 1.9-fold higher in seeds, coleoptiles and radicles, respectively.

**Fig. 4:**
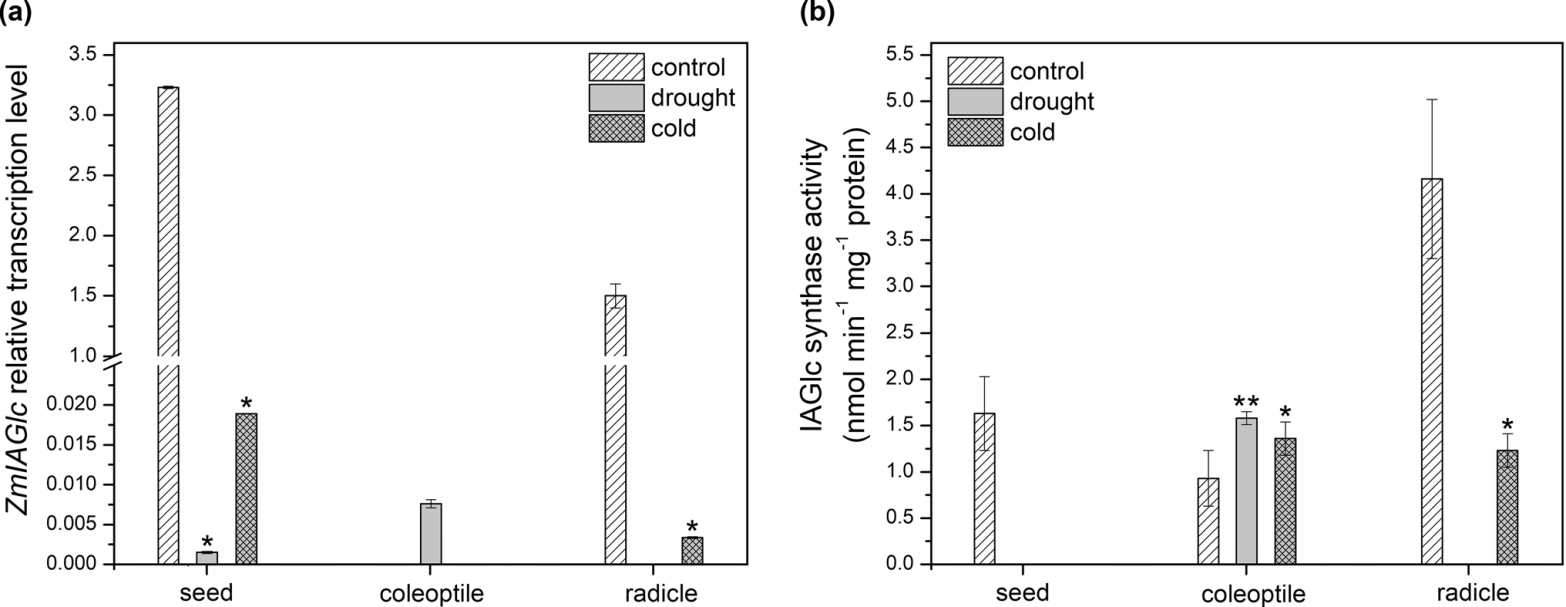
IAGlc synthase in 6-d-old maize seedlings subjected for 24 h to cold and drought stress. **a** Expression level of *ZmIAGlc* determined by quantitative RT-PCR analysis. **b** Enzymatic activity of IAGlc synthase. Data are expressed as mean ± SD for 3 biological repetitions of each assay (*n* = 3). Statistical significance is indicated by *, *P* < 0.05; **, *P* < 0.01 (*t*-test). No bar visible in the graph means there was no transcript or activity detected in the material. No asterisk means that statistical analysis could not be performed because control value was 0

Relative expression of *ZmIAGlc* is presented in Fig. 4a. In seeds, transcript level was the highest in control seedlings (3.23), while expression level decreased drastically to 1.5 × 10^–3^ under drought and 1.89 × 10^–2^ under cold stress. Similar pattern was observed for radicles, where *ZmIAGlc* transcript was 1.5 in control seedlings and 0 and 3.36 × 10^–3^ under drought and cold, respectively. Together with high transcript level, IAGlc synthase activity was also higher in control seeds and radicles (Fig. 4b). For seeds, the enzyme activity was detected only in control material. In radicles, no IAGlc synthase activity was present under drought, while in cold exposed seedlings activity of the enzyme was 3.4-fold lower compared to the control. In coleoptiles, *ZmIAGlc* transcript was detected only in drought-subjected seedlings (7.6 × 10^–3^). However, the enzyme was active in coleoptiles under all investigated conditions, with the highest activity reported for drought-exposed seedlings (Fig. 4b). The IAGlc activity was slightly lower in the control coleoptiles compared to the seedlings exposed to both stresses.

**Fig. 5:**
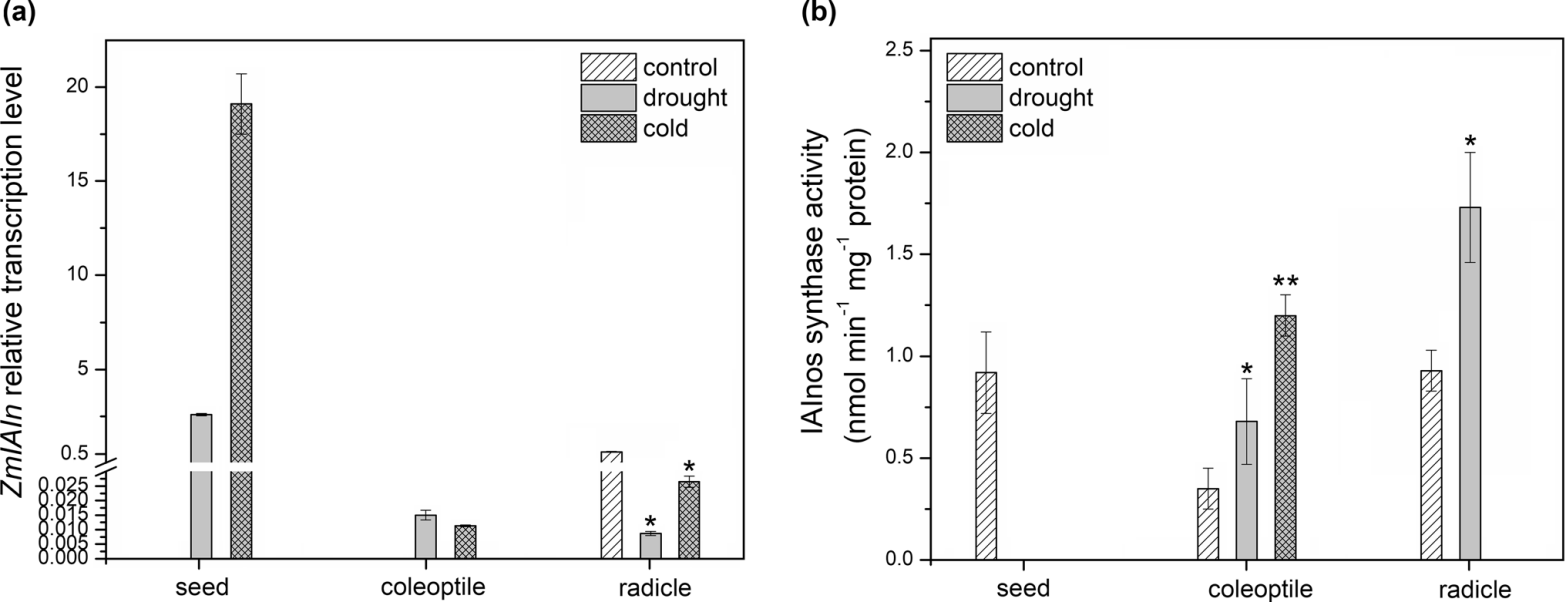
IAInos synthase in 6-d-old maize seedlings subjected for 24 h to cold and drought stress. **a** Expression level of *ZmIAIn* determined by quantitative RT-PCR analysis. **b** Enzymatic (IAInos biosynthesis) activity of IAInos synthase. Data are expressed as mean ± SD for 3 biological repetitions of each assay (*n* = 3). Statistical significance is indicated by *, *P* < 0.05; **, *P* < 0.01 (*t* test). No bar visible in the graph means there was no transcript or activity detected in the material. No asterisk means that statistical analysis could not be performed because control value was 0

Relative expression level of *ZmIAIn* and IAInos synthase activity is shown in Fig. 5a and b, respectively. No *ZmIAIn* transcript was detected in control seeds, while relative expression level was high in seeds of the seedlings grown under drought (2.6) and cold stress (19.1). On the other hand, the IAInos synthase was not catalytically active in seeds of the seedlings subjected to the stress but only in the control material despite absence of the transcript. In coleoptiles, transcript was also not detected in control material but its level in stress-subjected seedlings was much lower compared to the seeds, being 1.5 × 10^–2^ and 1.13 × 10^–2^ under drought and cold, respectively. Despite the low expression level, IAInos synthase was active in coleoptiles under all examined conditions, with the lowest activity in control material. In coleoptiles of the drought-subjected seedlings, enzyme activity increased 1.9-fold and in cold-treated seedlings, it increased 3.4-fold compared to the control. In the control radicles, *ZmIAIn* transcript level was 0.62 but decreased rapidly to 8.7 × 10^–3^ and 2.65 × 10^–2^ under drought and cold stress, respectively. As for IAInos synthase activity, it was completely diminished in radicles of cold-treated seedlings but increased 1.9-fold under drought compared to the control.

### Effect of IAInos on level of selected stress markers in maize seedlings

Exposition of maize seedlings to stress conditions results in changes in expression and activity of both IAGlc synthase and IAInos synthase. It shows that under abiotic stress IAInos synthesis pathway is regulated to manage IAA and/or IAInos concentration in the plant. As IAA is one of the most important plant growth regulators, modulation of its concentration is certainly a vital purpose of IAInos synthesis pathway as it allows plant adaptation to stressful environment. However, it is also possible that IAInos alone also plays a role in plant stress response as it is for another IAA conjugate, IAA-Asp (Ostrowski et al. 2016). To examine possible role of IAInos in plant stress response, we have analyzed effect of this conjugate on level of some stress markers, namely ascorbate concentration, protein carbonylation and lipid peroxidation. As IAInos is a hydrolysable conjugate, we have compared its effect with effect of IAA and *myo*-inositol, which are products of IAInos hydrolysis.

**Fig. 6:**
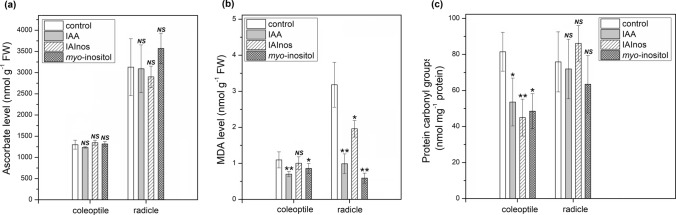
Effects of 10 μM IAA, 10 μM IAInos or 10 μM *myo*-inositol on stress markers in coleoptiles and radicles of 6-d-old maize seedlings. **a** Ascorbate concentration. **b** Level of malondialdehyde (MDA). **c** Level of carbonyl groups in proteins. Data are expressed as mean ± SD for 3 biological repetitions of each assay (*n* = 3). Statistical significance is indicated by *, *P* < 0.05; **, *P* < 0.01; *NS*, not significant (*P* > 0.05) (*t* test)

As shown in Fig. 6a, the ascorbate level was not affected by exogenous IAA, IAInos or *myo*-inositol, neither in coleoptiles or radicles of maize seedlings. IAInos also did not affect level of lipid peroxidation in coleoptiles of 6-d-old maize seedlings, however, MDA (malondialdehyde, product of lipid peroxidation) level was slightly diminished in coleoptiles of IAA (1.6-fold decrease) and *myo*-inositol (1.3-fold decrease) treated seedlings (Fig. 6b). However, in radicles, treatment with all examined compounds reduced lipid peroxidation with the highest decrease (5.4-fold) caused by *myo-*inositol and the slightest 1.6-fold decrease resulted from IAInos treatment.

The level of carbonyl groups in proteins is shown in Fig. 6c. Protein carbonylation was not affected by any of the tested compounds in maize radicles in a statistically significant manner. However, in coleoptiles of IAA, IAInos and *myo*-inositol treated seedlings, protein carbonylation was 1.6-fold, 1.8-fold and 1.7-fold diminished, respectively.

## Discussion

### Changes in IAInos biosynthesis pathway during maize seedling development

IAInos biosynthesis pathway analysis in the whole germinating seeds, as well as in the seedlings, revealed that during young maize development the IAA level undergoes changes. In the whole seeds, the free phytohormone level increased twofold in the transition between 3-d- and 6-d-old seedling. It is well known that IAA induces seeds dormancy and its level needs to be reduced in order to induce germination (Tuan et al. 2019). During germination, IAA is released from its conjugates and is supplied to the developing seedling (Bialek et al. 1992; Casanova-Sáez et al. 2021). This can possibly explain increase in IAA level in 6-d-old germinating maize seeds. During maize growth, expression profiles of *ZmIAGlc* and *ZmIAIn* in germinating seeds have also changed. The level of *ZmIAGlc* transcript increased ninefold, while transcription of *ZmIAIn* was completely diminished. However, despite changes in the expression of both synthases, the enzymes activities remained on a similar level in 6-d-old germinating seeds compared to the younger ones. Conjugation of IAA to glucose is the first step of phytohormone inactivation in IAInos biosynthesis pathway. Thus, it is possible, that a higher level of IAA in seeds of 6-d-old seedlings induced transcription of *ZmIAGlc* to reduce the level of this auxin. IAA was proven to induce expression of another IAA-glucosyltransferase gene, a rice *OsIAGLU* (He et al. 2020) in germinating rice seeds. It should be mentioned that the *ZmIAGlc* expression level was high not only in 6-d-old but also in 3-d-old germinating maize seeds. Similarly, *A. thaliana* uridine diphosphate glycosyltransferase (UGT) 75D1 gene (*UGT75D1*), which encodes another glucosyltransferase that conjugates auxins (mainly indole-3-butyric acid) to glucose, is strongly expressed in germinating seeds during the period of 3–14 days after germination (Zhang et al. 2016a). Moreover, elevated expression in germinating seeds was also reported for *OsIAGLU* (He et al. 2020). Lack of increase in IAGlc synthase activity may be due to the expression regulation on the translational level. It is also possible that other mechanisms of enzyme regulation are employed, such as regulation by post-translational modifications, as potential sites of *N*-glycosylation and phosphorylation are present in the protein sequence (Szerszen et al. 1994), or by activity modulators such as ATP or glucose-1-phosphate that affect IAGlc synthase activity in concentration dependent manner (Ciarkowska et al. 2021). As for IAInos synthase, we suppose that in 6-d-old germinating seeds transcription of *ZmIAIn* gets completely diminished to inactivate the second step of IAInos biosynthesis for IAA level to remain elevated. The IAInos synthase must still be present in the seeds, thus the activity of the enzyme is not yet affected in 6-d-old material. It is possible that at two examined stages of the seed (3-d- and 6-d-old), differently glycosylated isoforms of the IAInos synthase are present in cells. The glycosylation of IAInos synthase was previously suggested to act as a regulation mechanism of the enzyme activity in maize and rice (Kowalczyk et al. 2003; Ciarkowska et al. 2018). Interestingly, germinating seeds exhibit a different profile of the IAInos synthesis pathway than immature seeds from corn cobs, where the highest activities of both enzymes correspond to the strongest activity of L-Trp aminotransferase (TAA), which is responsible for de novo IAA biosynthesis (Ostrowski et al. 2020).

In coleoptiles of the 6-d-old maize seedlings IAA level, as well as expression of both synthases, decreased compared to the 3-d-old material, however, enzymatic activities were slightly elevated. Possibly, IAInos synthesis pathway becomes less relevant for IAA homeostasis in coleoptiles of older seedlings. Liu et al. (2019) reported that low expression level of auxin conjugating OsIAGT1/OsIAAGLU was characteristic also for young rice tissues. Complete loss of IAGlc and IAInos synthase transcription may suggest that other mechanisms depleting IAA level in coleoptiles must be active. Other possible explanation is that lower IAA concentration in coleoptiles results in suppressing the IAInos synthesis pathway as inactivation of the hormone is not needed or even could lead to growth retardment. In many transgenic plants which overexpress genes encoding glucosyltransferases conjugating auxins to glucose, the level of the phytohormone was reduced leading to inhibition of hypocotyls elongation. Such an effect was observed in *A. thaliana* overexpressing *UGT84B1* (Aoi et al. 2020) and rice overexpressing *OsIAGT1/OsIAAGLU* (Liu et al. 2019; Yu et al. 2019). Thus, in young coleoptiles, the IAA concentration must be maintained on a proper level by inactivation of conjugation process to ensure cell elongation.

It is widely known that shoots and roots indicate different requirements for auxin concentration during realization of their physiological events. So, a comparison of IAA conjugation in these plant organs could be interesting in the context of auxin homeostasis. In roots, high auxin concentration stimulates initiation of lateral roots but inhibits primary root elongation (Woodward and Bartel 2005). Thus, auxin level is one of main factors determining root architecture. Level of IAA in radicles of 6-d-old maize seedlings increased compared to 3-d-old material possibly as the primary radicle length in still young seedlings was not yet sufficient. Enzymatic activities of IAGlc and IAInos synthases were higher in the radicles of older seedlings what is not consistent with increased auxin level. Moreover, expression of *ZmIAGlc* and *ZmIAIn* was highly elevated in the older seedlings. Possibly, induction of expression of the genes resulted from high IAA level. It has been previously reported, that both genes are auxin regulated. Expression of *OsIAGT1/OsIAAGLU* is IAA induced (Yu et al. 2019). IAInos synthase activity was up-regulated in rice seedlings upon exogenous IAA treatment (Ciarkowska et al. 2016). It is also possible that other mechanisms may be responsible for elevated free IAA level as IAA ester conjugating enzymes activities are not always consistent with free auxin level. Transgenic tomato plants with decreased IAGlc synthase activity exhibited reduced free IAA level even though the opposite result was expected (Iyer et al. 2005). On the other hand, high free IAA level observed in 6-d-old maize radicles may also be a result of high IAGlc synthase activity. UGT74D1 over-expressing *A. thaliana* mutants exhibited elevated free IAA level caused by alteration of expression of auxin-related genes: up-regulation of auxin synthesis *YUC* genes and down-regulation of auxin influx carrier *AUX* (Jin et al. 2021)*.*

Changes in IAInos synthesis pathway between 3-d-old and 6-d-old maize seedlings and germinating seeds indicate a role of this pathway in regulation of IAA level during development of maize at the early growth stage. However, discrepancies between auxin level and changes in IAGlc and IAInos synthases expression and activity strongly suggest participation of other mechanisms in managing IAA level in young maize, e.g. auxin amide conjugates synthesis as such a pathway is also present in maize (Feng et al. 2015; Zhang et al. 2016b).

### Changes in IAInos biosynthesis pathway in maize seedling under drought and cold stress

Environmental stress, such as drought and cold, affects plant growth and thus causes lower crop productivity (Xiong et al. 2002). Plants have developed numerous mechanisms to help them cope with the stress. One of stress adaptation strategies is managing local concentrations of IAA in plant by alteration of auxin metabolism and transport (Potters et al. 2007).

In germinating seeds of 6-d-old maize seedlings exposed for 24 h to drought or cold, transcript level of *ZmIAGlc* drastically dropped, followed by complete loss of IAGlc synthase catalytic activity. IAInos synthase also lost its enzymatic activity under stress. High level of transcript may indicate that *ZmIAIn* gene expression was activated to supplement IAInos synthase as the enzyme is not active under stress conditions. IAInos biosynthesis pathway plays a significant role in management of IAA concentration in germinating seeds under drought, as total abolishment of both synthases activities resulted in almost twofold increase in free auxin concentration. The role of this pathway under cold stress seems less relevant as IAA concentration remains on the same level as in control seeds. On the contrary, exogenous abscisic acid (ABA) which is the most important phytohormone related to stress response, increased transcription of rice *OsIAGLU* in germinating seeds (He et al. 2020). Though in this case, ABA may not have acted as a mediator of stress response but rather as germination regulator, as ABA is one of phytohormones responsible for managing this process (Shu et al. 2016). In case of maize germinating seeds some mechanisms, other than ABA signaling, may be involved in lowering expression of *ZmIAGlc*.

Similar to the germinating seeds, in coleoptiles of 6-d-old maize seedlings exposed to cold stress, the IAA level remained unchanged but was elevated in seedlings subjected to drought. In cold-treated material, coleoptiles exhibited increased IAGlc and IAInos synthesizing catalytic activities. It cannot be excluded that both synthases act in a temperature-dependent manner as level of UGT76F1, a glucosyltransferase from *A. thaliana*, which conjugates indole-3-pyruvic acid to glucose, is regulated transcriptionally depending on temperature to control hypocotyl growth (Chen et al. 2020). There may be other mechanisms involved in maintaining auxin level as unchanged concentration of IAA under cold compared to the control is not in consistency with increased IAA-ester conjugating enzymes activities. Possibly, enzymes from GH3 family participate in auxin level mediation in response to cold stress in maize. It has been previously reported that GH3 amidosynthetases play a role in rice response to cold as plants overexpressing *OsGH3-2* exhibited increased tolerance to this stress due to decrease in free IAA concentration (Du et al. 2012). As shown by Pavlović et al. (2018), ester and amide conjugation of IAA can be regulated differently under stress conditions.

In coleoptiles of drought exposed maize seedlings, expression and activity of IAGlc and IAInos synthase were elevated, possibly to cope with increased free IAA level. Overexpression of *UGT74E2* resulted in better drought tolerance of *A. thaliana* (Tognetti et al. 2010). Similar results were found for *UGT75D1* overexpressing *A. thaliana* seedlings exposed to mannitol-induced osmotic stress (Zhang et al. 2016a).

In radicles of drought exposed maize seedlings, transcription of *ZmIAGlc* and *ZmIAIn* was completely halted and strongly reduced, respectively. IAGlc synthase activity was also entirely diminished resulting in an almost twofold increase in IAA level. On the other hand, IAInos synthase activity increased in drought affected radicles. Possibly, under drought conditions, synthesis of IAInos synthase is up-regulated until depletion of *ZmIAIn* transcript. Another possible explanation is that IAInos synthase activity is regulated post-translationally by covalent modifications or presence of activators. Then, activity of the enzyme could be elevated despite low transcript level. However, elevated IAInos synthase activity has no significant effect on the IAA level as a substrate of the enzyme, 1-*O-*IAGlc, cannot be supplied by inactive IAGlc synthase. As auxin accumulation in *A. thaliana* roots upon abiotic stress results in inhibition of root elongation (Liu et al. 2016), it is likely that increase in IAA level mediated by modulation of IAInos synthesis pathway in drought exposed maize seedlings is an important stress-adaptive mechanism. In the radicles of cold affected maize seedlings, the expression of both synthases is also down-regulated; however, under such stress, IAInos synthase is already inactive while IAGlc synthase activity is only decreased. As IAA concentration in radicles of cold exposed seedlings was maintained on similar level compared to the control, it suggests that under stress conditions IAGlc synthase has more important role than IAInos synthase in inactivating IAA by its conjugation.

Altogether, the above-described results suggest that IAInos biosynthesis pathway has a role in regulation of free IAA concentration during stress response in maize, however, other pathways regulating auxin level must also be at play as changes in free IAA concentration are not always convergent with IAA-ester conjugating enzymes activities.

### Effect of IAInos on level of selected stress markers in maize seedlings

We have determined that IAInos biosynthesis pathway plays a role in plant stress response. However, it is not clear whether this role is restricted to control of free IAA level or maybe IAInos as a molecule has an individual function in stress response. Thus, we analyzed the effect of IAInos on selected stress markers. Moreover, we have compared its effect with the effect of IAA and *myo*-inositol, products of IAInos hydrolysis, as both compounds are known for having a role in stress response (Irvine and Schell 2001; Potters et al. 2007). It should be noted that studies by Jakubowska et al. (1993) have shown that IAInos is a poor source of free IAA for the growth of oat (*Avena sativa)* coleoptiles. In addition, the purified bifunctional IAInos synthase/IAInos hydrolase from maize liquid endosperm displayed significantly lower hydrolytic activity in comparison to IAInos synthesis (Kowalczyk et al. 2003). IAInos hydrolysis was also observed in homogenate from immature seeds of pea (Jakubowska and Kowalczyk 2004). Thus, we cannot exclude that IAInos delivers IAA and *myo*-inositol for the modulation of plant responses to stress.

Under stress conditions, the level of reactive oxygen species in a plant increases causing oxidative damage to molecules such as lipids and proteins (Meitha et al. 2020). Simultaneously, antioxidative mechanisms are promoted to prevent such damage. In effect, under stress, level of non-enzymatic antioxidants, such as ascorbate, usually is elevated. To examine the possible role of IAInos in plant stress response we have analyzed the effect of this conjugate, as well as IAA and *myo*-inositol, on level of ascorbate concentration, protein carbonylation and lipid peroxidation.

Exogenous IAInos had no effect on ascorbate level in maize seedlings, it also did not affect lipid peroxidation in coleoptiles or protein carbonylation in radicles. However, IAInos decreased lipids peroxidation in radicles and protein carbonylation in coleoptiles. Nevertheless, both IAA and *myo*-inositol had either similar or stronger effect. One of the possible explanations is that the observed effect of IAInos may have resulted from its hydrolysis to IAA and *myo*-inositol. As such, this auxin conjugate has no proven individual role in plant stress response modulation but only affects it by managing the concentration of free IAA and *myo*-inositol. Thus, so far IAA-Asp remains the only auxin conjugate with a reported direct role in plant stress responses (Ostrowski et al. 2016). These results suggest that the IAInos biosynthesis pathway may control IAA concentration during the developmental processes as well as responses to environmental conditions. However, in contrast to an amide conjugate, IAA-Asp, it seems to be questionable that IAInos itself can modulate the stress response.

## Concluding remarks

IAInos biosynthesis pathway which leads to temporal IAA inactivation plays a role in managing auxin concentration during maize seedling development. It also has a function in maize response to drought and cold stress that is restricted to controlling free IAA and *myo*-inositol level. Maintenance of auxin homeostasis is a complex process involving numerous mechanisms. Thus, it should be noted that the IAInos biosynthesis is only one of the mechanisms that regulate free IAA concentration under such conditions, as also biosynthesis, degradation, transport and conjugation to other kinds of molecules can modulate local auxin level.

### *Author contribution statement*

AC: project administration and funding acquisition. AC and MO: conceptualization, formal analysis, and supervision. AC, MO, and JK: methodology, resources, and writing—original draft preparation. AC, MO, PW, and JK: investigation. AC, MO, and PW: writing—review and editing. All authors have read and agreed to the published version of the manuscript.

## Data Availability

The authors declare that all data generated during this study are included in the manuscript.

## Abbreviations

GH3: Gretchen Hagen 3
IAA-Asp: Indole-3-acetyl-aspartate
IAGlc: 1-*O*-Indole-3-acetyl-β-D-glucose
IAInos: Indole-3-acetyl-*myo*-inositol
UGT: Uridine diphosphate glycosyltransferase
*ZmIAGlc*: IAGlc synthase encoding gene
*ZmIAIn*: IAInos synthase encoding gene
